# Unveiling Adulterated Cheese: A ^1^H-NMR-Based Lipidomic Approach

**DOI:** 10.3390/foods14162789

**Published:** 2025-08-11

**Authors:** Maria-Cristina Todașcă, Mihaela Tociu, Fulvia-Ancuța Manolache

**Affiliations:** 1 Faculty of Chemical Engineering and Biotechnology, National University of Science and Technology Politehnica Bucharest, 1-7 Polizu Street, 011061 Bucharest, Romania; mihaela.tociu@upb.ro; 2 National Research & Development Institute for Food Bioresources—IBA, 5 Ancuta Baneasa Street, 020323 Bucharest, Romania; fulvia.manolache@bioresurse.ro

**Keywords:** cheese, fatty acid profile, NMR, adulteration

## Abstract

The main objective of this research consists in finding a rapid method for cheese lipidomics based on NMR data. This study plays an important role in differentiation and characterization of cheese samples in accordance with fat composition, especially in the case of fat substitution with exogenous animal or vegetal fat. Our findings play an important role in relation to religious requirements regarding non-allowed foods (pork fat, for example, in some cultures) and in the correct characterization of foods according to their lipidic profile. The approach consists in establishing a fingerprint region (0.86–0.93 ppm from ^1^H-NMR spectra) and then creating a database of the results obtained. The evaluation of the long-chain saturated fatty acids and the saturated short-chain fatty acids (C4 to C8) was established with a newly developed set of equations that make the computation possible even when mixtures of fats from different sources are present. This was accomplished by developing a new method for quantification of the fatty acid composition of different types of cheese, based on ^1^H-NMR spectroscopy. Principal component analysis (PCA) was applied to 40 cheese samples with varying degrees (0%, 5%, 12%, or 15%) of milk fat substitution (pork fat, vegetable fat, hydrogenated oils) and different clotting agents (calcium chloride or citric acid). The best sample discrimination was achieved using fatty acid profiles estimated from ^1^H-NMR data (using a total of six variables), explaining 89.7% of the total variance. Clear separation was observed between samples containing only milk fat and those with added fats. These results demonstrate that the integration of ^1^H-NMR spectroscopy with principal component analysis (PCA) provides a reliable approach for discriminating cheese samples according to their fat composition.

## 1. Introduction

Milk and dairy products represent a major segment of the agri-food industry, primarily due to their complex nutritional profile, being rich sources of bioavailable lipids, fat-soluble vitamins, and essential micronutrients [1,2]. Cheese is a fresh or matured dairy product obtained through milk coagulation, characterized by its complex matrix of proteins, short-chain fatty acids, vitamins, and minerals, which contribute to its high nutritional value [3].

Food authenticity, particularly for products susceptible to adulteration such as dairy products, edible oils, and wines, remains a significant focus of scientific investigation [4,5]. In the case of dairy products, adulteration often involves the partial or complete substitution of milk fat with lower-cost alternatives, including vegetable oils or non-dairy animal fats, primarily for economic gain [6]. Economically motivated adulteration represents a persistent challenge in the food industry, undermining both product integrity and consumer confidence. This reality emphasizes the urgent need for effective protective measures to safeguard consumers. Accordingly, the development of rapid, cost-efficient, and robust analytical methods for detecting fraudulent practices in commercial food products has gained increasing attention [1,7,8].

Food quality and safety remain central concerns for consumers globally. To ensure consumer safety, robust food safety measures in cheese production are practiced, as well as continuous vigilance in monitoring and controlling the microbiota [9]. Due to their widespread consumption and economic value, dairy products are particularly prone to adulteration [10]. This susceptibility underscores the necessity for the development of robust, rapid, and accessible analytical tools for product authentication. A wide range of analytical methods have been employed to determine the fatty acid profile of dairy products, including gas chromatography (GC), Fourier-transform infrared (FT-IR) spectroscopy [11], visible–near-infrared reflectance spectroscopy [12], near-infrared spectroscopy (NIR) [13,14], atomic absorption spectroscopy, UV–visible spectrophotometry [15], fluorescence spectroscopy [16], and nuclear magnetic resonance (NMR) spectroscopy. When combined with chemometric analysis, these techniques have demonstrated significant potential for food authentication and quality control [11,17,18]. Among these, GC is the most widely used technique for the analysis of fatty acids in cheese lipids [11,19,20]. Despite its high sensitivity and established reliability, GC is often criticized for being a time-consuming, labor-intensive, and expensive technique [21,22]. Unlike other techniques, NMR spectroscopy has the added feature of lower environmental impact, meaning it contributes to chemistry’s “green” transition [23].

The necessity of this research arises from multiple factors. From a nutritional perspective, the integrity of milk fat in cheese plays a crucial role in determining its nutritional value, particularly due to its unique composition of short- and medium-chain fatty acids, essential for human metabolism. The substitution of milk fat with lower-quality or non-dairy fats can alter the nutritional profile of cheese, potentially impacting consumer health. Furthermore, safeguarding consumers against fraudulent practices is crucial for ensuring transparency within the food supply chain and upholding trust in labelling accuracy and established quality standards [24]. Adulteration not only misleads consumers but also poses risks to individuals with dietary restrictions or allergies.

Cheese adulteration involves a variety of fraudulent practices that differ in complexity, health risk, and economic effect. These methods are primarily used to lower production costs, enhance product yield, or replicate the characteristics of premium or Protected Designation of Origin (PDO) cheeses. A quantitative analysis of related studies showed that species substitution was the most studied type of adulteration, accounting for 41.3% of studies. This was followed by fat substitution (21.2%), protein adulteration (13.5%), the addition of non-dairy additives (11.5%), and geographic mislabeling (7.7%). These statistics highlight the industry’s focus on identifying animal-species fraud and alterations in fat profiles, especially in high-value PDO cheeses [25].

Proton nuclear magnetic resonance (^1^H-NMR) spectroscopy offers several advantages, including rapid analysis, non-destructive measurements, and minimal sample preparation [26,27]. This method has proven effective in the analysis of complex lipid mixtures such as fats and oils [28], butter and margarine [29], and in the detection of butter adulteration with lard [30], as well as for overall food quality assessment [31].

^1^H-NMR-based metabolomics, through the characterization of polar and lipid fractions, has proven effective in differentiating traditional cheeses, such as Greek Graviera, according to geographical origin and authenticity, while its combination with multivariate analysis enabled the discrimination of PDO Grana Padano from non-PDO cheeses by providing a robust metabolomic fingerprint [32,33].

The objective of this study is to develop a rapid and reliable method for the detection of cheese adulteration with exogenous fats of animal or vegetable origin using ^1^H-NMR spectroscopy. This technique enables the accurate characterization of cheese lipid profiles and offers the advantages of speed and minimal sample preparation.

## 2. Materials and Methods

Laboratory-prepared cheese samples were analyzed to determine their fatty acid composition. Based on these analyses, a characteristic spectral fingerprint region was identified, indicative of the origin of the fat present in the samples. Furthermore, the NMR spectral data were subjected to multivariate statistical analysis, specifically principal component analysis (PCA), to differentiate authentic milk-based cheese from imitation products formulated through the substitution of milk fat with pork fat, palm oil, or other types of exogenous fat.

### 2.1. Samples

A total of 40 samples of fresh cheese were obtained in the laboratory from pasteurized cow’s milk standardized at 1.5% fat content (skimmed milk). The milk samples were standardized with exogenous fat in different proportions from 0% up to 15%, using 4 types of fat: milk fat, palm fat, pork fat (lard), and hydrogenated oil. For each sample, 500 mL of milk was heated to 80°C and the caseinates were precipitated by adding different clogging agents like citric acid (10 mL of citric acid solution at a 10% concentration) or calcium chloride salt (1 mL of calcium chloride solution at a 10% concentration). The samples were left to precipitate while cooling down for 2 h and were then filtered for 12 h. Thus, the 40 samples of cheese obtained in the laboratory with various added fats and clogging agents are presented in Table 1. All cheese samples were obtained in triplicate.

The last column on Table 1 corresponds to cheese samples that were obtained by adding exogenous fat consisting of a mixture of all 4 fat types. Therefore, equal amounts (in weight) of milk fat, hydrogenated oil, palm oil, and pork fat were added to the cheese samples to reach the desired proportion of fat.

### 2.2. Dry Matter Content

All cheese samples were characterized regarding their dry matter content. A measure of 30 g of each sample was heated in a laboratory oven to 103 ± 2 °C in order to evaporate the residual moisture (mostly water). The remaining solid residue was subsequently weighed, and the dry matter content was calculated as a proportion of the initial mass, in accordance with the standardized method SR 1286:1997.

### 2.3. Total Fat Content

The dried cheese samples were subjected to lipid extraction using petroleum ether as the solvent, following a continuous Soxhlet extraction procedure for 6 h, in accordance with the standardized method SR EN ISO 659:2009 [34].

### 2.4. ^1^H-NMR Spectroscopy

The ^1^H-NMR spectra of the fats extracted from the cheese samples were recorded on a Bruker Avance III 400 spectrometer, operating at 9.4 Tesla, corresponding to a resonance frequency of 400.13 MHz for the ^1^H nucleus, and equipped with a direct detection four-nuclei probe head and field gradients on the z axis. Typical parameters for ^1^H-NMR spectral acquisition from Bruker TopSpin (1D sequence using 30-degree flip angle, zg30, v 1.11.6.1) were used. The acquisition parameters used were 90° high-power pulse, 3.98 s acquisition times, 8.2 KHz spectral window, 16 scans, and 0.25 Hz fid resolution. No FID processing was conducted prior to Fourier transformation. The average acquisition time of the ^1^H-NMR spectra was approximately 2 min. Sample preparation was carried out directly on the fat extracted from cheese, without any prior derivatization, allowing analysis in its native triacylglycerol form. A volume of 0.2 mL fat was dissolved in 0.8 mL of deuterated chloroform (CDCl_3_), and the resulting mixture was transferred into 5 mm NMR tubes (Wilmad 507) for spectral acquisition. All spectra were processed with TopSpin 4.0.7. software. The chemical shift was measured based on the internal standard TMS (tetramethylsilane).

### 2.5. PCA

The chemometric analysis of the data was performed using the XLStat 2023 software (developed by Addinsoft, Paris, France). Principal component analysis was conducted on the NMR data (integral values) and also on the fatty acid composition calculated for each sample based on the ^1^H-NMR data.

## 3. Results

**Figure 1:**
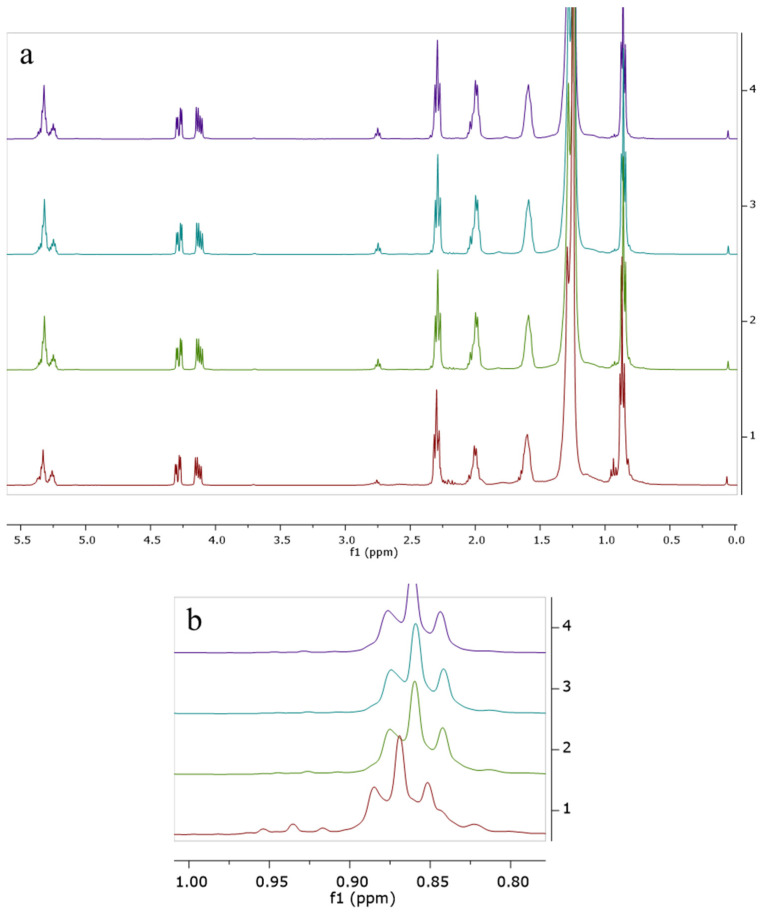
^1^H-NMR spectrum of cheese samples with added palm fat, using calcium chloride as clogging agent (1—POCa1—0% added palm oil, 2—POCa2—5% added palm oil, 3—POCa3—12% added palm oil, 4—POCa4—15% added palm oil). (**a**) General profile, 5.5–0 ppm; (**b**) detailed area, 0.75–1 ppm.

The lipid fractions extracted from all 40 cheese samples were analyzed using proton nuclear magnetic resonance (^1^H-NMR) spectroscopy to determine their compositional profiles. Figure 1 shows the superimposed spectra of the fat extracted from the cheese sample with different amounts of added palm oil and precipitated with calcium chloride.

The general NMR profile of the fats extracted from the cheese samples is presented in Figure 1. Several differences are visible in the ^1^H-NMR profile of the extracted fat. For instance, in the case of the added palm oil, the intensity of the signal at 2.8 ppm is increasing, with the signal being generated by the bis-allylic protons (mainly from linolenic and linoleic acids), which are characteristic of vegetable unsaturated fats [35]. The most important area of the spectrum is the region between 0.75 and 0.1 ppm. In the mentioned region of the spectra, there are two important triplets: one at 0.93 ppm, generated by the methyl groups from the saturated short-chain fatty acids (C4–C8) characteristic of milk fat, and another at 0.86 ppm, generated by the methyl group from the saturated and unsaturated long-chain fatty acids (>C8). Therefore, this region is very important for this study because it could ease the separation of the samples with added palm oil since the cheese obtained with milk fat will be the only one where the triplet from 0.93 ppm is present. As can be seen in the detailed area in Figure 1b, with the increase in added palm oil fat in the cheese sample, the signal generated by the short-chain saturated fatty acids characteristic of milk fat is decreasing and becomes lost in the noise when 10% or more palm oil is added to the cheese samples.

**Figure 2:**
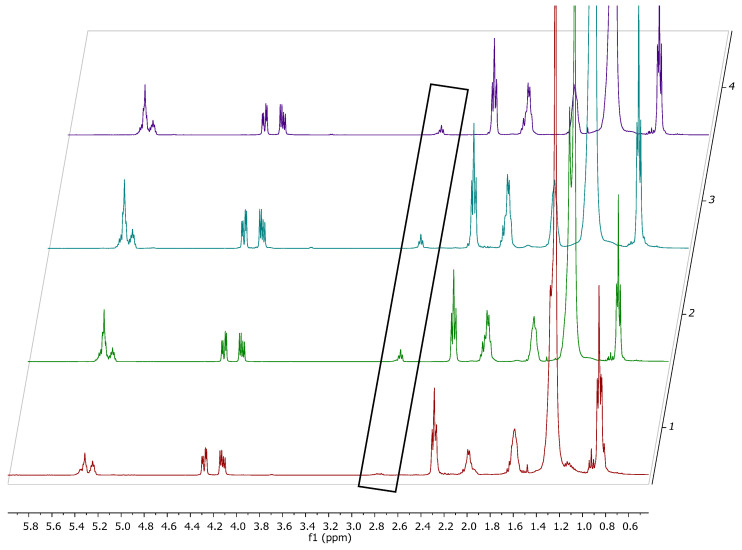
^1^H-NMR spectrum of the fat extracted from the cheese samples with 12% added fat: 1—MFCa3 (milk fat), 2—PFCa3 (pork fat), 3—POCa3 (palm oil), 4—HOCa3 (hydrogenated vegetable oil).

A similar situation was noticed when other types of fat were used to replace the milk fat in the cheese samples. In Figure 2, the general profile of the fat extracted from cheese samples obtained with 12% added fat of different types (milk fat, pork fat, palm oil, and hydrogenated vegetable oil) is shown. As can be seen, the signal at 2.85 ppm is well represented in all samples except the one obtained with milk fat, since this signal is characteristic of long-chain unsaturated fatty acids (bis-allylic protons, mainly from linolenic and linoleic acids), which are not characteristic of the fat extracted from cow milk.

Religious and ethical considerations play an equally critical role in the context of food authenticity. For individuals adhering to specific dietary frameworks—such as Halal, Kosher, or vegetarian diets—the undeclared presence of animal-derived fats, including lard, represents a significant infringement on their dietary restrictions and belief systems. Consequently, the development and implementation of rapid, reliable screening techniques such as ^1^H-NMR are essential to strengthen food traceability, safeguard consumer rights, and ensure conformity with nutritional, regulatory, and ethical standards.

**Figure 3:**
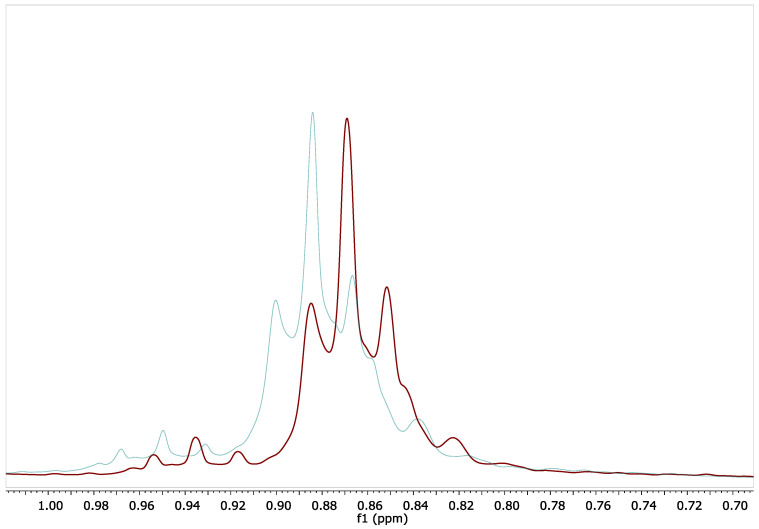
Detailed area of the ^1^H-NMR spectrum of the fat extracted from the cheese samples with added palm fat using different clogging agents: calcium chloride in red (POCa1) and citric acid in green (POC1).

The precipitation agent shows no influence on the fat cheese composition; only small differences in the dry matter content and in the total fat content of the obtained samples are distinguished. In Figure 3, a slight shift of the signals towards the left is noticed due to the small pH change.

**Figure 4:**
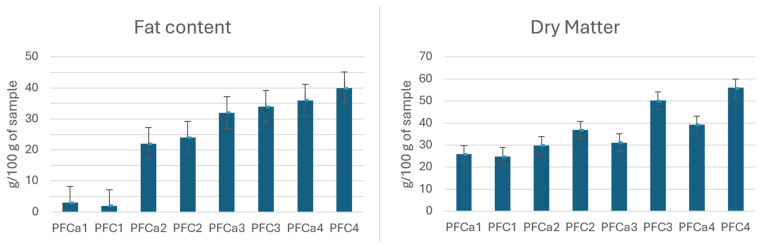
Mean values for dry matter and total fat content of the cheese samples obtained with added pork fat, using calcium chloride (Ca) and citric acid (C) as clogging agents.

There are some slight differences between the samples regarding dry matter and total fat content in correlation with the precipitation agent used and the exogenous fat used in substitution. For example, in Figure 4, the fat content and dry matter are presented for the cheese samples obtained with pork fat as the additional fat and with calcium chloride (Ca) and citric acid (C) as clogging agents. As can be seen, when citric acid is used, the dry matter and the total fat content of the cheese samples are higher than in the case of samples obtained with calcium chloride precipitation.

In a previous study, [5], we established a fast method for the differentiation of dairy samples obtained only from milk, based on an indicator calculated as the ratio between long-chain and short-chain fatty acids, calculated based on ^1^H-NMR spectroscopy data. The set of equations used for the quantification of the fatty acids from the NMR spectra can be applied only in the case of samples that contain one type of fat (either animal fat or vegetable fat). In the case of mixtures of fats from different sources, the clear evaluation of the fatty acid content regarding the main classes (saturated, monounsaturated, polyunsaturated) becomes more challenging due to signals overlapping in the 0.9–1 ppm area of the ^1^H-NMR spectrum. Therefore, in this study, we have developed a method for the precise evaluation of fatty acid content according to the main classes of fatty acids, which can be used to detect the substitution of milk fat with exogenous fat and to estimate the quantity of the substitution as well.

**Figure 5:**
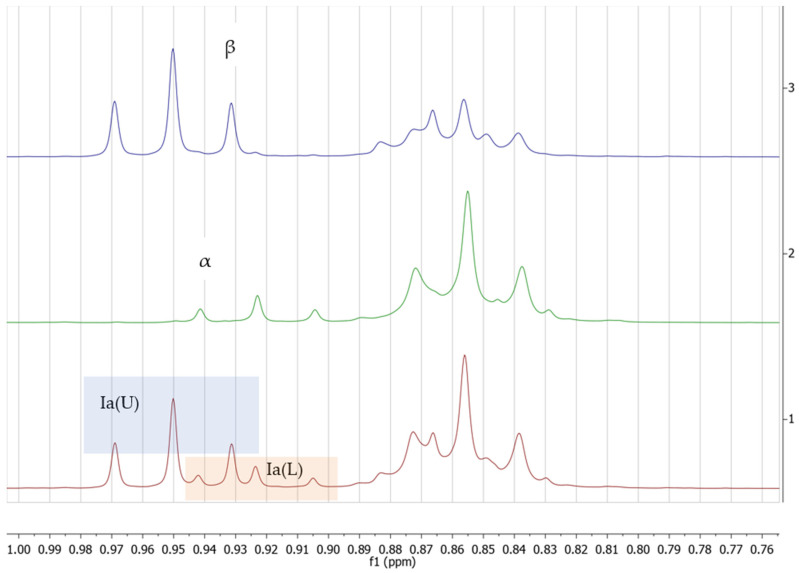
Detailed area from 0.75 to 1 ppm of the ^1^H-NMR spectrum for: 1—a mixture of milk fat and linseed oil, 1:1 (*w*:*w*); 2—milk fat; 3—linseed oil.

A model was created based on 10 mixtures with different proportions of milk fat and linseed oil. In Figure 5, the detailed area of the ^1^H-NMR spectrum shows two important triplets in the area 0.9–1 ppm. The triplet marked with the orange rectangle corresponds to the terminal -CH_3_ groups from short-chain fatty acids (C4 to C8) characteristic of milk fat, while the triplet marked with the green rectangle corresponds to the terminal -CH_3_ groups from polyunsaturated long-chain fatty acids (PUFA), from the linseed oils, which are characteristic of vegetable oils. The corresponding integral values were marked with Ia(U) for the methyl group from PUFA and Ia(L) for the short-chain saturated fatty acids.

**Figure 6:**
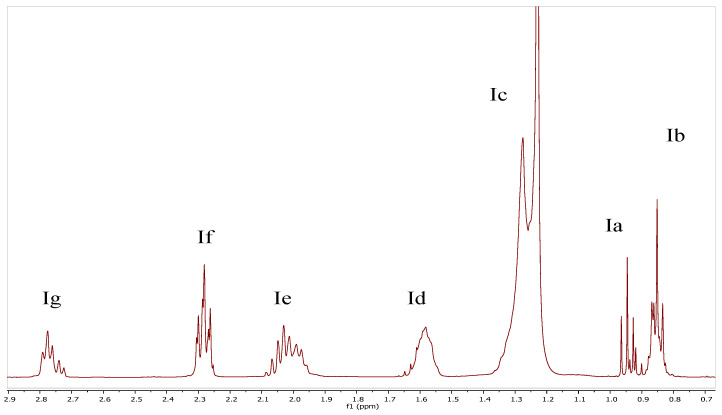
Area between 0.7 and 2.9 of the ^1^H-NMR spectrum for a mixture of milk fat and linseed oil, 1:1 (*w*:*w*), where Ia and Ib are generated by the terminal -CH_3_ groups, Ic is generated by the -CH_2_ groups, Id is generated by the protons of the β position to the carbonyl group, Ie is generated by the allyl protons (all unsaturated fatty acids), If is generated by the protons of the α position to the carbonyl group, and Ig is generated by the bis-allylic protons, mainly from linolenic and linoleic acids.

Considering all the above information and the corresponding integral values (as shown in Figure 6), a new version of the equations are proposed for the quantitation of the fatty acids of fat extracted from cheese samples with different exogenous fats:

(1) $$k=\frac{Ia(U)-Ia(L)+Ib}{3}$$

(2) $$x=\frac{Ia(U)-\alpha }{3\cdot k}$$

(3) $$y=\frac{3\cdot Ig-4\cdot (Ia(U)-\alpha )}{6\cdot k}$$

(4) $$z=\frac{3\cdot Ie-6\cdot Ig+4\cdot Ia(U)-\alpha }{12\cdot k}$$

(5) t=1−x−y−z

(6) $$s=\frac{Ia(L)-\beta }{3\cdot k}$$

where k is a constant, s is short-chain saturated fatty acids, x is polyunsaturated fatty acid, y is monounsaturated fatty acids, z is di unsaturated fatty acids, t is saturated fatty acids, and α and β correspond to the value of the integral, measured only on one pick of the triplet corresponding to methyl groups (see Figure 5).

The ^1^H-NMR spectral data were subjected to principal component analysis (PCA), and the results show good differentiation of the milk cheese from adulterated ones. The control cheese (marked with 1) contains only fat from milk, either from the skimmed milk used for the cheese production or from milk fat addition in various proportions, without any addition of exogenous fats clustered together. The cheeses with added exogenous fat tend to cluster in a separate region with no important separation related to the amount of substitution or the type of exogenous fat added.

**Figure 7:**
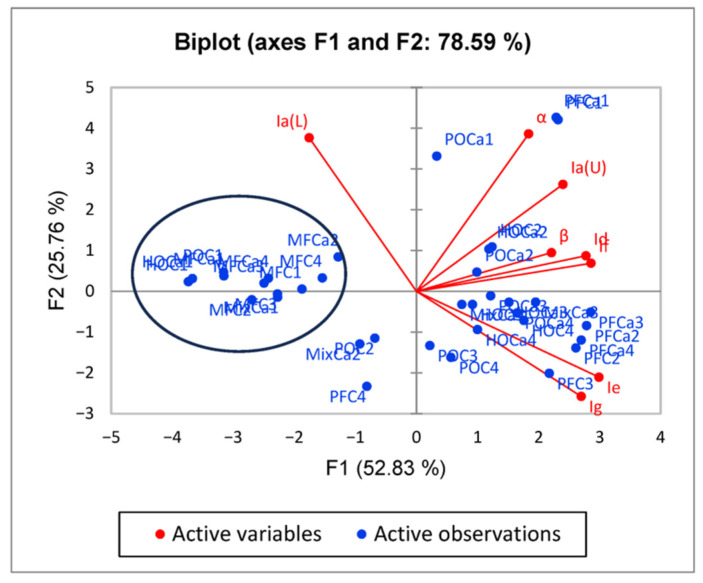
Authentication of milk cheese versus cheese substitute obtained with different exogenous fats based on integral values from ^1^H-NMR spectra. Ia and Ib: terminal -CH3 groups; Ic: -CH2 groups; Id: protons of the β position to the carbonyl group; Ie: allyl protons (all unsaturated fatty acids); If: protons of the α position to the carbonyl group; Ig: bis-allylic protons (mainly from linolenic and linoleic acids); Ia(U): methyl group from PUFA; Ia(L): short-chain saturated fatty acids.

In Figure 7, the integral values are used in the PCA, resulting in a good separation of the samples. The tendency of the cheese samples with milk fat to cluster in the same region is visible (please see the circled area), but no important separation of the samples associated with the precipitation agent used in the cheese production can be seen.

**Figure 8:**
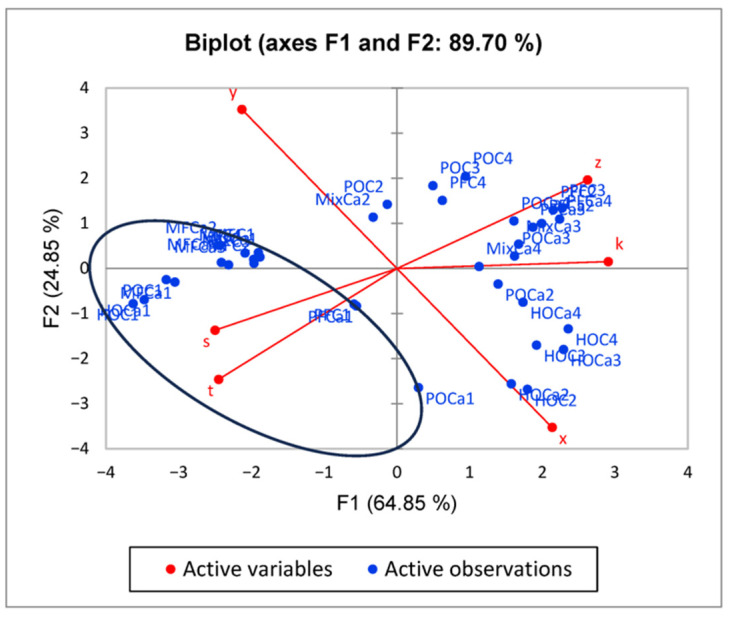
Authentication of milk cheese versus cheese substitute obtained with different exogenous fats based on indicators obtained using the described equations on ^1^H-NMR data. k: constant; s: short-chain saturated fatty acids; x: polyunsaturated fatty acid; y: monounsaturated fatty acids; z: diunsaturated fatty acids; t: saturated fatty acids.

Better results in terms of sample discrimination (the variability of the system is 89.7%) are obtained when the data used for the chemometrical analysis are the fatty acid composition values estimated based on the ^1^H-NMR data using the equations described above. In Figure 8, the clear separation of the cheese obtained only with milk fat is observed (all samples containing only milk fat are marked with a circle), while the samples containing added pork and palm fat tend to cluster in a different area than the samples that were obtained with vegetable hydrogenated oils. Therefore, in this case, even a separation of the samples in accordance with the exogenous fat added is possible, since the samples with the addition of pork fat and palm oil are clustered in the first cartesian quadrant (positive values of both principal components), while the samples with the addition of hydrogenated oil are clustered in the forth cartesian quadrant (negative values of F1 and positive values of F2).

The repeatability of the milk fat analysis using the ^1^H-NMR method was evaluated. The relative standard deviation (RSD) values ranged from 0.92% to 2.37% across all analyzed peaks, and the corresponding standard deviation (SD) values were between 0.034 and 0.05. The reproducibility values ranged from 0.07 to 2.09. For milk fat dissolved in deuterated chloroform, the limit of detection (LOD) and limit of quantification (LOQ) were determined to be 3.34 g/L and 3.42 g/L, respectively. Based on these parameters, the estimated measurement uncertainty for all analyzed ^1^H-NMR signals was in the range of 0.01 to 0.08.

If we consider the feasibility of the proposed method, NMR spectroscopy analyzes the distinct spectral patterns of adulterants in adulteration detection regarding different cheeses. Differences in chemical shifts, peak intensities, and coupling constants can be identified by comparing the NMR spectra of authentic and adulterated samples. This allows for the quantification and identification of added vegetable oils with high accuracy. Moreover, it offers non-destructive analysis, requiring minimal sample preparation, as is mentioned in the literature [36]. Nevertheless, the NMR method has historically been considered an expensive method; nowadays, this has become less important when the reduced instrumental time, sample preparation and fast analytical time are considered, alongside the method’s vast application in cheese quality control, composition quantification, authentication of origin, ripening monitoring, and flavor development [37].

## 4. Conclusions

A new set of equations have been established, based on ^1^H-NMR spectral data, which offers good results in terms of fatty acid quantification in cheese samples regarding five important classes of fatty acids: PUFA, DUFA, MUFA, and SFA with long chains and short chains (C4–C8). The procedure is fast and reliable with important benefits, especially due to recent benchtop NMR development, which provides the means for handy operation.

The quantification method could be easily adapted for quality control of other types of dairy products, where the extraction of the fat could be carried out and analyzed based on the presented results. Preliminary studies on hard cheese, fermented cheese, and vegetable hard cheese (obtained from vegetable substitutes for animal milk) make us believe that good differentiation of the samples in accordance with the exogenous fat added or present in the specific product could be achieved. The method shows potential for application to complex food matrices that contain both fat from milk and from other sources (vegetable or animal fat). Many traditional food products and newly developed ones (even functional foods) contain mixtures of fats from different sources that up till now could raise difficulties when the type of fatty acids contained by the extracted fat needed to be quantified by means of ^1^H-NMR spectroscopy. This enables both scientists working in lipidomics and industrial producers to facilitate quality control and authentication of various classes of foods.

## Figures and Tables

**Table 1:** Cheese samples obtained in the laboratory with added exogenous fats in different proportions using enzymatic precipitation.

Proportion of the Added Fat	Milk Fat		Hydrogenated Oil		Palm Oil		Pork Fat		Mixture of Exogenous Fats	
0%	MFCa1	MFC1	HOCa1	HOC1	POCa1	POC1	PFCa1	PFC1	MixCa1	MixC1
5%	MFCa2	MFC2	HOCa2	HOC2	POCa2	POC2	PFCa2	PFC2	MixCa2	MixC2
12%	MFCa3	MFC3	HOCa3	HOC3	POCa3	POC3	PFCa3	PFC3	MixCa3	MixC3
15%	MFCa4	MFC4	HOCa4	HOC4	POCa4	POC4	PFCa4	PFC4	MixCa4	MixC4
Precipitation agent	Calcium chloride	Citric acid	Calcium chloride	Citric acid	Calcium chloride	Citric acid	Calcium chloride	Citric acid	Calcium chloride	Citric acid

## Data Availability

The original contributions presented in the study are included in the article, further inquiries can be directed to the corresponding author.
